# Does Informal Caregiving Lead to Parental Burnout? Comparing Parents Having (or Not) Children With Mental and Physical Issues

**DOI:** 10.3389/fpsyg.2018.00884

**Published:** 2018-06-06

**Authors:** Pierre Gérain, Emmanuelle Zech

**Affiliations:** ^1^National Fund for Scientific Research, Brussels, Belgium; ^2^Person Centered Research and Training Lab, Psychological Sciences Research Institute, Université catholique de Louvain, Louvain-la-Neuve, Belgium

**Keywords:** burnout, exhaustion, parent, informal caregiver, family caregiver, children, personality, special needs

## Abstract

**Introduction:** Parenting a child with special needs (CSN) may be an important challenge. Previous research has highlighted an increased risk of parental burnout among parents caring for their CSN. Yet, these studies only focused on children with specific issues and did not consider the wide variety of CSN. There is thus a need to take a more global approach to assessing the impact of caring for a CSN on parental burnout. In addition, the impact on parental burnout of personality and parenting (dis)agreement needs to be measured to have a better understanding of parent-caregivers’ (PCgs) burnout.

**Method:** An online survey was completed by a large sample of parents from which a subsample of PCgs was identified.

**Results:** *T*-tests highlighted significantly more parental burnout among parents of CSN. However, further analyses showed that parents with only one child with one special need did not experience significantly more burnout than parents with typical children. The significant difference lay in the presence of comorbidity or the presence of multiple CSN in the family. Hierarchical regressions showed an important impact of Neuroticism for every burnout facet, along with co-parenting (dis)agreement. Subjective consequences of having to care for a CSN were also related to the burnout facets of both emotional exhaustion and emotional distancing.

**Discussion:** The presence of comorbidity and of multiple CSN in the family were related to more PCg burnout, emphasizing the need to consider these situations in further research. The role of neuroticism in PCg burnout confirms previous research both in parental and professional contexts. Parenting (dis)agreement also highlights the importance of dyadic support among parents. Finally, the importance of subjective aspects suggests that parental perception of their situation remains a central element in understanding the consequences of caregiving.

## Introduction

Although one of the most positive experiences reported by parents, parenting might also be hazardous. Recent research has shown that some factors might lead to a form of burnout among parents (Mikolajczak et al., 2017). Parental burnout could be defined as a syndrome in reaction to parental stress. It is composed of emotional exhaustion (EE) (its core component), depersonalization/emotional distancing (ED), and lack of personal accomplishment (LPA). A growing literature investigates parental burnout but a particular situation is the experience of parents caring for a child with a chronic illness (Norberg and Green, 2007) or a disability (Basaran et al., 2013). This situation appears to put parents caring for a child with physical or mental issues at greater risk of burnout. These studies converge with a wide literature on taking care of relatives – children or not – exploring the risk of burnout among informal or family caregivers. However, most research on informal caregivers recruit participants based on the specific illness of the child or the relative concerned (e.g., diabetes mellitus, autism, cancer). This approach fails to give a transversal understanding of PCgs’ burnout. To this end, a more global approach is needed to consider and assess the impact of having a child with specific needs on parental burnout.

Informal caregivers are often family members, typically unpaid, providing care to someone with whom they have a personal relationship (Schulz and Tompkins, 2010). By definition, all parents are therefore informal caregivers because they all take care of dependent relatives, namely, their child(ren). However, a distinction is made due to the extra care needed by children with special needs (CSNs). Those children present special health needs, defined in terms of use of services, therapies, counseling, medication, or functional limitations that last for at least a year (Schulz and Tompkins, 2010). These needs include a wide range of issues causing activity limitations, from emotional or learning disabilities, to physical illnesses or disabilities. Parents taking care of a CSN could therefore be defined as PCgs’ – as opposed to parents of “typical” children.

To date, several studies have investigated whether PCg are more prone to experiencing feelings of burnout than parents with typical children. A recent meta-analysis on informal caregivers’ burnout collected every study comparing caregivers with a control group on burnout (Gérain and Zech, unpublished). The vast majority of these studies focused on PCg of children with various special needs – from brain cancer survivors (Lindahl Norberg, 2007) to children with autism (Weiss, 2002). A summary effect size was computed and yielded a medium difference of burnout between PCg and parents of typical children (*d* = 0.46). Thus, parents caring for a CSN experience more burnout than other parents.

However, all the studies on PCg burnout have proceeded in a similar way, that is, they selected a sample of CSN and then compared it with a convenient control sample. This approach may impact studies’ outcomes because of recruitment bias for both the control and the PCg group. For the control group, the selection might be biased by the convenient nature of the sample. Often, little is known about how (i.e., on which criteria) the group was selected, suggesting that the control sample may not have been selected as rigorously as the PCg sample. For the PCg group, the risk might be to select parents in specific contexts, thus inflating (or deflating) the occurrence of burnout symptoms among the sample. Selecting children with a particularly acute illness is more likely to result in parents with higher burnout scores. A complementary approach would be to use a sample of parents from which parents with a CSN would be selected. This approach – which is closer to the population-based approach (though still economical) – would make it possible to select the parents of CSN who would not otherwise be reached by a PCg-centered study. This approach would perfectly complement previous research on PCg by providing a different perspective of data on parental burnout symptoms. By shifting the focus from the child to the parents, new kinds of PCg could appear, such as parents taking care of more than one CSN. This would also allow to investigate the impact on PCg burnout of having to care for one or several children and having not only one but multiple issues.

Beyond these questions there is still a need to understand the risk factors of burnout for PCgs. To date, no specific personality trait variable has been investigated among informal caregivers, except for alexithymia (Katsifaraki and Wood, 2014) and Performance Based Self-Esteem (Lindström et al., 2011). The potential role of Big-Five personality traits in the onset of burnout symptoms still needs to be examined. Neuroticism (the reverse of emotional stability) appears as a stable risk factor of burnout both in professional and parental contexts (Alarcon et al., 2009; Mikolajczak et al., 2017). This tendency to experience more emotional instability toward negative feelings might also play a role in PCg burnout. However, there is still a lack of studies supporting such a hypothesis and little is understood about how personality traits might impact burnout in such a context.

Parenting a child often takes place in a dyadic parenting context. Direct indicators of the importance of a good relationship with the other parent have already been shown as protective factors of parental burnout (Lindström et al., 2011). One study showed that feeling the spouse’s lack of interest regarding the care of the common CSN also represented a risk of burnout for the PCg (Demirhan et al., 2011). In addition to the quality of the inter-parental relationship, the importance of having a common view about the future of the child could play a role in the development of burnout symptoms. Such an indicator has been described as co-parenting (dis)agreement (Feinberg et al., 2012), i.e., the degree to which parents agree on issues related to raising their child (Mendez et al., 2015). Disagreement in this area could represent a supplementary challenge when having a CSN in as much as one PCg does not feel supported by the other parent, and thus feels the lack of a common vision about important decisions regarding their child’s special needs (Mendez et al., 2015).

Being a PCg can also be associated with several perceived detrimental consequences. Previous studies have documented some of these, such as the perception that the illness affects everyday life and induces sleep disruptions (Lindström et al., 2011). Additional consequences could also be explored such as the perception of having been obliged to give up important things due to having a CSN, or a perceived disruption of the family organization.

In sum, the present research sets three different investigation goals to be explored in a large sample of parents: (1) to compare parents having a CSN with parents with no-CSN; (2) to understand which caregiving setting is more at risk of burnout between having one CSN, multiple CSN or a CSN with comorbidities; and (3) to assess the impact of descriptive variables, neuroticism, co-parenting disagreement and the perceived impacts of having a CSN.

## Materials and Methods

The present database has also been used in an article on the validation of the Parental Burnout Assessment (Roskam et al., 2018). This study was carried out in accordance with the recommendations of APA ethical standards, Ethics Commission of the Psychological Sciences Research Institute (Université catholique de Louvain). The protocol was approved by the Ethic Commission of the Psychological Sciences Research Institute (Université catholique de Louvain). All subjects provided informed consent in accordance with the Declaration of Helsinki.

### Sample and Recruitment

A total of 900 parents was recruited for the present study, plus one parent excluded because he did not report having a child. Among the sample, 647 (71.9%) were English-speaking and 253 (28.1%) were French-speaking. A majority of participants were from England (53.3%), Belgium (26.3%), and the United States (9.6%). Other participants were from Canada and several European countries. Descriptive information about the entire sample is presented in **Table 1**.

**Table 1:** Descriptive variables of the sample of parents with and without children with special needs (CSNs).

Variable		Parents without CSN	Parents with CSN	Total
		(*N* = 725)	(*N* = 175)	(*N* = 900)
Age	Mean	36.31	38.38	36.71
	SD	6.71	7.12	6.84
	Range	[20–59]	[21–57]	[20–59]
Gender	Male	160 (22.1%)	24 (13.7%)	184 (20.4%)
	Female	565 (77.9%)	151 (86.3%)	716 (79.6%)
Working status	Full-time	326 (45.0%)	64 (36.6%)	390 (43.3%)
	Part-time	238 (32.8%)	45 (25.7%)	283 (31.4%)
	Homeworker or without work	161 (22.2%)	66 (37.7%)	227 (25.2%)
Marital status	Biparental couple	639 (88.1%)	144 (82.3%)	783 (87.0%)
	Solo parent	86 (11.9%)	31 (17.7%)	117 (13.0%)
Education	Secondary school or less	295 (40.7%)	90 (51.4%)	385 (42.8%)
	Bachelor degree	247 (34.1%)	56 (32.0%)	303 (33.7%)
	Master degree	129 (17.8%)	26 (14.9%)	155 (17.2%)
	Post-master degree	54 (7.5%)	3 (1.7%)	57 (6.3%)
# children	Mean	1.97	2.65	2.1
	SD	0.88	1.20	1.03
	Range	[1–7]	[1–7]	[1–7]
Age children	Mean	8.29	11.38	9.04
	SD	6.67	7.01	6.88
	Range	[0–39]	[0–39]	[0–39]
Gender Children	Male	674 (50%)	201 (46.3%)	875 (49.1%)
	Female	674 (50%)	233 (53.7%)	907 (50.9%)

The study was labeled as “Being a parent in the 21st century” (Eng) or “*Être parent aujourd’hui*” (Fr). English-speaking parents were recruited through Prolific, a platform to recruit subjects for scientific online research (Palan and Schitter, 2017). French-speakers were recruited through social networks, websites, forums, and snowball effect. The French speakers also had the opportunity to participate in a lottery to win €200 by providing their email address in a separate questionnaire.

Every respondent was asked if each of their children presented a form of (1) chronic or serious disease, (2) disability, or (3) behavioral, emotional, or learning disorder. For each participant and for each type of issue, participants answered by Yes/No if their child presented one or more of these issues. Therefore, each child of each participant was profiled through these three characteristics. Parents reporting at least one of their children having at least one of the three issues were categorized as parents of a CSN, a PCg. Parents not reporting such issues among any of their children were categorized as parents of typical children. Information about these subsamples is also presented in **Table 1**.

### Measures

#### Socio-Demographic Measures

The participants had to provide their age, gender, education level, number of children, and marital status. For each child, they had to provide the age and gender as well as information about whether the child lived at home or away.

#### Parental Burnout

The Parental Burnout Inventory^1^ (PBI; Roskam et al., 2017) is a 22-item self-report measurement of parental burnout. It was adapted from the Maslach Burnout Inventory – Human Services Survey (MBI, Maslach et al., 1996). The PBI distinguishes three parental burnout dimensions: EE, ED, and Personal Accomplishment. The EE subscale is based on 8 items (e.g., “*I feel emotionally drained by my parental role*”) and assesses the feeling of the respondent as being emotionally drained by the parenting role. The ED describes a colder relationship with the children, a progressive lack of attention and emotional expression toward them. This subscale is constituted of 8 items (e.g., “*I am less attentive to my children’s emotions*”). Personal accomplishment refers to a feeling of low competence and achievement in the parental role. It is assessed with 6 items (e.g., “*I accomplish many worthwhile things as parent*”). This latter subscale is reversed in order to obtain a score of LPA with higher scores indicating higher symptoms of parental burnout. Each item is rated on a 7-level Likert scale from 1 (“*Never*”) to 7 (“*Every day*”). In the present study, the PBI had an excellent internal consistency on each subscale (α = 0.936 for EE, α = 0.919 for ED, α = 0.837 for the LPA).

#### Neuroticism

The Neuroticism subscale of the Big Five Inventory (BFI; John et al., 1991; Plaisant et al., 2010) was used in order to evaluate Neuroticism. This subscale includes 8 items starting with “*I am someone who* …” followed by the statement (e.g., “*is moody, has up and down mood swings*,” or “*worries a lot*”). Respondents were asked to refer not especially to their current state but rather how they are in general (i.e., their nature). They had to answer with 5-level Likert scales ranging from 1 (“*Strongly disagree*”) to 5 (“*Strongly agree*”). Items are both positively and negatively phrased. Positively phrased items were recoded to obtain a homogeneous measure. The Cronbach alpha in the present study was good (α = 0.824).

#### Co-parenting (Dis)Agreement

The Agreement subscale of the Co-Parenting Scale (Feinberg et al., 2012) was used to assess the co-parenting (dis)agreement. It consists of 4 items (e.g., “*My partner and I have the same goals for our child(ren)*”) rated on a 7-point Likert-scale from 1 (“*not at all true for us*”) to 7 (“*absolutely true for us*”). Scores were reversed in order to obtain a composite score of co-parenting disagreement. The Cronbach alpha in the present study was good (α = 0.828).

#### Perceived Psychosocial Impact of Having a CSN

Parents having a child with CSN were also asked five exploratory Yes/No binary questions regarding the impact of having a child with issues: (1) “My child’s disease/disability/disorder has a strong impact on our daily routine,” (2) “Because of his/her disease/disability/disorder, my child requires constant care or attention,” (3) “I feel constantly preoccupied by my child’s disease/disability/disorder.” (4) “Because of my child’s disease/disability/disorder, I had to give up things that were important to me (work and/or activities, etc.),” (5) “Because of my child’s disease/disability/disorder, I had to give up my life.” The impact of each variable was evaluated separately due to the diversity of the elements.

#### Non-included Measures

The present study also included other measures that were not used for the present report. A new measure of parental burnout, the PBA, was submitted in order to compare it with the psychometric properties of the PBI. This data is analyzed in the PBA validation paper (Roskam et al., 2018). A measure of family disorganization (CHAOS) was also included. Finally, and only in the English-speaking sample, a measure of Job Burnout (MBI) was also used.

### Data Analysis

The IBM SPSS Statistics software was used to analyze the data. Group comparisons were performed through ANOVAs and *post hoc* Hochberg GT2 analyses were added due to large differences in group sizes (Field, 2013). Pearson correlations were performed between all variables of interest. Correlation are presented as supplementary material (see SM1).

Hierarchical linear regression analyses were used to assess the impact on explained variance of the addition of variables sets. The first “descriptive” variable set was constituted of descriptive variables on the parent and the family (i.e., gender, age, education, working status, marital status, number of children). The second set, “parenting,” was constituted by co-parenting disagreement. The third set, “personality,” was constituted by neuroticism. The fourth set, “CSN descriptive,” was constituted by descriptive variables regarding the parent-caregiving context (i.e., presence of multiple CSN in the family, and whether the CSN presented any comorbidity). Finally, the fifth set, “Perceived Impact,” was constituted by variables regarding the perceived psychosocial impact of having a CSN.

## Results

A total of 901 people completed the questionnaire. One parent did not report having any children and was excluded from the data.

### Comparing Parents With Typical Children and Parents With Children With Special Needs

Parents reporting having a least one child with a CSN were compared on burnout measures to parents reporting having no child with special needs. For EE, parents with CSN reported significantly more symptoms (*M* = 17.70, *SD* = 12.84) than parents with no CSN (*M* = 12.81, *SD* = 10.88), *t*(889) = -4.63, *p* < 0.001, Cohen’s *d* = 0.43. For ED, parents with CSN also significantly scored higher (*M* = 7.91, *SD* = 9.45) than parents of no CSN (*M* = 5.42, *SD* = 6.82), *t*(889) = -3.28, *p* = 0.001, Cohen’s *d* = 0.34. For LPA, significance indicators were slightly lower but a comparable pattern remained. Parents with CSN had more symptoms of LPA (*M* = 8.35, *SD* = 6.84) than parents with no CSN (*M* = 7.00, *SD =* 6.51), *t*(889) = -2.42, *p* = 0.016, Cohen’s *d* = 0.20.

### Comparing Caregiving Settings

Parents reporting having (or not) a CSN were then categorized into four groups regarding the specific characteristic(s) of their children’s special needs. The groups were constituted of parents reporting having no children with any of the surveyed issues (*n* = 725), having one child with one issue (*n* = 98), having a child with multiple issues (comorbidity) (*n* = 35), and having multiple children with one or more issues (*n* = 42).

To assess the difference in burnout symptoms between parents with no CSN with PCg, three ANOVAs were computed for each burnout subscale (see **Table 2**). For the EE subscale, a significant group effect was highlighted, *F*(3,887) = 13.70, *p* < 0.001, η^2^ = 0.04. *Post hoc* analyses are reported in **Table 2**. These analyses showed no difference between parents of children with no issues and parents with one child having one issue (*p* = 0.158, Cohen’s *d* = 0.24). However, the former group was significantly different from the group of parents with one child with comorbidity (*p* < 0.001, Cohen’s *d* = 1.01) and from parents having more than one CSN (*p* = 0.038, Cohen’s *d* = 0.44). On ED, a group effect was also found, *F*(3,887) = 7.72, *p* < 0.001, η^2^ = 0.03. A comparable pattern was found, *post hoc* analyses showing that parents of one CSN were not different from parents with no CSN (*p* = 0.618, Cohen’s *d* = 0.17). Parents with no CSN reported significantly less ED than parents of a child with comorbidities (*p* = 0.005, Cohen’s *d* = 0.62) and parents of multiple CSN (*p* = 0.003, Cohen’s *d* = 0.57). Finally, for LPA, a group effect was also highlighted, *F*(3,887) = 3.35, *p* = 0.019, η^2^ = 0.01. However, *post hoc* analyses only showed a significant difference between parents of no CSN and parents of multiple CSN (*p* = 0.035, Cohen’s *d* = 0.44), the latter having a greater LPA than the former. Thus, except for this latter burnout subscale, a pattern appears with parents of no CSN not having significantly less EE and ED symptoms than parents of one CSN. However, they reported significantly less burnout symptoms than parents of a child with comorbidity or parents having multiple CSN.

**Table 2:** Comparison of parental burnout scores of parents with or without children with special needs (CSN).

	Typical (*n* = 717)		One CSN (*n* = 97)		CSN with comorbidity (*n* = 35)		Multiple CSN (*n* = 42)		*F-*test	partial η^2^
	M	SD	M	SD	M	SD	M	SD		
EE	12.81^a^	10.88	15.47^ab^	11.87	23.89^c^	12.61	17.67^bc^	13.75	13.70***	0.04
ED	5.42^a^	6.82	6.58^ab^	7.73	9.74^b^	10.36	9.48^b^	11.77	7.72***	0.03
LPA	7.00^a^	6.51	7.48^ab^	6.08	8.89^ab^	7.08	9.88^b^	8.06	3.35*	0.01

*EE, emotional exhaustion; ED, emotional distancing; LPA, lack of personal accomplishment. ^∗^*p* < 0.05. ^∗∗^*p* < 0.01. ^∗∗∗^*p* < 0.001. ^*a-c*^Means with different superscripts within rows are significantly different at *p* < 0.05 when compared with the Hochberg GT2 *post hoc* tests.*

### Comparison of Risk Factors for Parents With or Without CSN

Pearson correlations were performed separately for parents with no CSN and for parents with a CSN (see Supplementary Material, SM1). Comparisons of correlation coefficients between the two groups were performed through the Fisher r-to-z transformation (Cohen, 1988). Only one correlation coefficient was significantly different between the no-CSN and the CSN groups (i.e., working fulltime is more associated to EE for parents with a CSN than parents with no-CSN, respectively, *r(174) =* -0.25, *p* = 0.001 and *r(717) =* -0.08, *p* = 0.034, *F* = 2.03, *p* = 0.021).

All the variables of interest were also computed for each group in a hierarchical linear regression to identify stronger indicators of burnout symptoms. All regressions yielded significant conclusions, with adjusted R^2^ ranging from.11 to.39 (see **Table 3**). The impact of each variable is presented in **Table 4** (for the complete model, see Supplementary Material (SM2) for the successive steps).

**Table 3:** Final regression models for the prediction of burnout subscales.

Group	EE		ED		LPA	
	*F*	Adj R^2^	*F*	Adj R^2^	*F*	Adj R^2^
Parents with no CSN	25.46^∗∗∗^	0.24	10.71^∗∗∗^	0.11	15.01^∗∗∗^	0.15
*F*(9,706)						
Parents with CSN	7.83^∗∗∗^	0.39	2.38^∗∗^	0.11	2.74^∗∗∗^	0.14
*F*(16,157)						

*EE, emotional exhaustion; ED, emotional distancing; LPA, lack of personal accomplishment. ^∗∗^*p* < 0.01. ^∗∗∗^*p* < 0.001.*

**Table 4:** Hierarchical stepwise linear regression model for the prediction of emotional exhaustion, emotional distancing, and lack of personal accomplishment.

Variable		EE			ED			LPA		
		β	*t*	ΔR^2^	β	*t*	ΔR^2^	β	*t*	ΔR^2^
Parents with no CSN^a^				0.24^c^			0.11^c^			0.15^c^
Step 1	Male	0.03	0.81	0.04***	0.07	1.86^†^	0.02*	0.13	3.44***	0.03***
	Age	–0.13	–3.92***		0.03	0.89		0.13	3.50***	
	Education	0.04	1.05		0.15	3.95***		0.07	1.92^†^	
	Solo parent	–0.03	–0.73		0.04	1.02		0.02	0.58	
	Full-time worker	–0.06	–1.26		0.02	0.32		–0.01	–0.26	
	Part-time worker	–0.07	–1.64^†^		0.03	0.56		–0.03	–0.64	
	#Children	0.06	1.66^†^		0.06	1.68^†^		0.02	0.65	
Step 2	Co-parenting disagreement	0.14	3.81***	0.05***	0.08	2.06*	0.02***	0.08	2.21*	0.02***
Step 3	Neuroticism	0.42	12.03***	0.15***	0.30	7.98***	0.08***	0.34	9.43***	0.11***

Parents with CSN^b^				0.39^c^			0.11^c^			0.14^c^
Step 1	Male	0.14	2.11*	0.13***	0.14	1.74^†^	0.05	0.15	1.85^†^	0.08*
	Age	–0.08	–1.22		–0.02	–0.28		0.11	1.49	
	Education	0.06	0.86		0.10	1.22		–0.02	–0.29	
	Solo parent	–0.03	–0.40		–0.02	–0.25		0.15	1.87^†^	
	Full-time worker	–0.09	–1.12		0.02	0.16		0.02	0.17	
	Part-time worker	0.02	0.34		0.04	0.47		–0.02	–0.23	
	#Children	0.04	0.54		–0.04	–0.40		0.13	1.52	
Step 2	Co-parenting disagreement	0.16	2.42*	0.05***	0.13	1.69^†^	0.03*	0.23	2.96**	0.06*
Step 3	Neuroticism	0.37	5.42***	0.12***	0.19	2.31*	0.04**	0.28	3.48***	0.07***
Step 4	CSN has a comorbidity	0.10	1.58	0.05***	0.05	0.63	0.02	0.00	–0.03	0.00
	Multiple CSN	–0.09	–1.33		0.02	0.26		0.01	0.13	
Step 5	Impact on family	0.15	2.35*	0.10***	0.02	0.22	0.06*	–0.04	–0.56	0.01
	Permanent attention	0.06	0.87		0.03	0.38		0.00	–0.03	
	Preoccupation due to issue	0.13	1.98*		0.11	1.45		0.03	0.43	
	Give up important things	0.17	2.26*		0.15	1.69^†^		–0.02	–0.19	
	Give up life	0.05	0.75		0.11	1.34		0.10	1.25	

*^*a*^*n* = 715. ^*b*^*n* = 173. ^*c*^Adjusted R^*2*^ of the model. EE, emotional exhaustion; ED, emotional distancing; LPA, lack of personal accomplishment. ^†^*p* < 0.10. ^∗^*p* < 0.05. ^∗∗^*p* < 0.01. ^∗∗∗^*p* ≤ 0.002.*

#### Descriptive Variables

The descriptive variables appeared to significantly add explained variance for each burnout subscale (from ΔR^2^ = 0.02 to 0.13). For EE, being a male had a detrimental impact only for parents with CSN (β = 0.14, *p* = 0.037) whereas it had no impact for the no-CSN parents (β = 0.03, *p* = 0.417). Conversely, age had an impact only for parents without CSN (β = -0.13, *p* < 0.001), older parents reporting less EE. No other descriptive variables had a significant impact on EE. For ED, higher education appeared as a risk factor only among parents without CSN (β = 0.15, *p* < 0.001). The more highly educated the parents, the more they reported being distant from their typical children. The descriptive bloc had no impact on ED among parents with CSN. For LPA among parents without CSN, older age, being male, and higher education appeared as risk factors. Among parents with a CSN, being male also tended to be a risk factor (β = 0.15, *p* = 0.067) but there was no significant impact of age and education. However, it appeared that marital status tended to contribute to a risk of LPA, solo parents reporting more LPA (β = 0.15, *p* = 0.063).

#### Co-parenting Disagreement

For every burnout facet and for parents both with or without a CSN, the addition of co-parenting disagreement appeared to explain significantly more variance of burnout facets (from ΔR^2^ = 0.02 to 0.06). For ED, although there was only a tendency among parents with CSN (β = 0.13, *p* = 0.092), the addition of the variable still led to significantly higher explained variance (ΔR^2^ = 0.03, *p* = 0.028). This thus confirms that parents who disagree about the education and care of their children are more prone to burnout symptoms, regardless of whether they have typical or untypical children.

#### Neuroticism

Like co-parenting disagreement, neuroticism appeared as a stable risk factor of each facet of burnout for parents both with or without CSN. Neuroticism was in particular a good predictor of EE for parents without CSN (ΔR^2^ = 0.15, *p* < 0.001) and for parents with CSN (ΔR^2^ = 0.12, *p* < 0.001).

#### CSN Descriptive Variables

Descriptive variables about the CSN added explained variance only for EE (ΔR^2^ = 0.05, *p* = 0.002) but neither for ED, nor for LPA. None of the variables constituting these predictors appeared significant, suggesting that, contrary to previous results, comorbidity or the presence of more than one CSN could partially explain more EE but not more emotional distance or LPA.

#### Perceived Psychosocial Impact

The perceived psychosocial impact explained 10% of additional variance of EE (ΔR^2^ = 0.10, *p* < 0.001) and 6% of ED (ΔR^2^ = 0.06, *p* = 0.037). No further variance was explained for LPA (ΔR^2^ = 0.01, *p* = 0.807). For EE, three variables appeared significant: perceiving that having a CSN changed the daily routine of the family (β = 0.15, *p* = 0.020), feeling constantly preoccupied by the issue of the child (β = 0.13, *p* = 0.050), and having to give up important things due to the issue of the child (β = 0.17, *p* = 0.025). For ED, only the latter tended to have a significant impact (β = 0.15, *p* = 0.093).

## Discussion

### Comparing Parents With or Without CSN

The present study aimed to compare the importance of parental burnout symptoms for parents having (or not) a child with special needs (CSN). The results indicated that the three dimensions of burnout symptoms were related to having a CSN. This concurs with previous literature that highlighted that parents with a CSN experience consistently more burnout than parents with no CSN (Gérain and Zech, unpublished). When examining parents’ caregiving situation more specifically, it appeared that this excess in burnout symptoms was due either to having to care for a child with a comorbidity and thus dealing with more than one issue, or when parents had more than one child with one or more special needs.

Interestingly, no significant difference was found between parents having no child with an issue and parents who had to deal with only one child with only one issue. This seems to disconfirm previous studies that focused on one specific issue or illness. This non-significant effect could be explained by the difference in recruitment strategies between the present study and previous ones. Here, the focus on a large unspecified sample of parents could reach parents that may have been overlooked in previous studies. Indeed, in those studies, parents were recruited because they had children with a specific illness or disability (such as diabetes, cancer) so parents at greater risk might be preselected. Here, respondents were not preselected based on the issue of the child nor on the PCg status of the parent. We were thus able to reach a wide variety of situations from light to heavy contexts of care, allowing to investigate the continuum of special needs and stressors that PCg face. The nature of such issues has yet to be defined, as well as defining what constitutes the difference with more burdensome issues.

This study is the first to show that having several CSNs is especially detrimental for parental burnout symptoms. This result may highlight the cumulative burden of having to care for multiple CSN. Even if having a single child with one issue may be as demanding as caring for a child with no CSN, having to deal with more than one issue may imply a new set of stressors for the parent and may also impact the whole family. Caring for one child is time-and- resources-consuming, but having more than one child to care for or more than one issue to deal with seems to consume even more time and more resources. Subjectively, it could be overburdening to learn to cope with two different issues and this could then lead to burnout symptoms. These hypotheses should further be investigated in the future.

In the present study, the most striking result lies in the impact of comorbidity. It is the first study to show the impact of comorbidity on PCg burnout with effect sizes ranging from very large to small (Cohen’s *d* = 1.012 for EE, Cohen’s *d* = 0.617 for DP and Cohen’s *d* = 0.288 for LPA). Previous studies on PCg burnout focused on one specific mental or physical illness. Yet, among CSNs, comorbidity is often present (e.g., Gabis et al., 2015; Flood et al., 2016). Though only comorbidity between broad categories of issues (i.e., disability, illness, and emotional/learning issues) was considered, these results contribute to the growing consideration of how comorbidity may affect caregivers’ life and well-being. For example, caregivers of elderly people were found to experience more difficulties when their relative presented an additional mental or physical health issues (Dauphinot et al., 2016). It seems that parents are no exception to this finding.

### Risk Factors of Parental Burnout

The present study also aimed at delineating the risk factors of parental burnout among PCg. Several variables appeared as stable predictors of parental burnout for all parents. Consistently with the broader literature on burnout, both neuroticism and co-parenting disagreement were found to be significant predictors of PCg burnout. First, neuroticism appeared a risk factor for both parents with or without CSN. In the present results, it was also the strongest predictor for almost every burnout facet (with ΔR^2^ ranging from 0.04 to 0.16). This result is the first to highlight the role of one Big-Five personality dimension on PCgs’ burnout. It echoes findings in the existing literature, where neuroticism has been found to be a fertile ground for increased prevalence of psychopathology and the occurrence of burnout symptoms (Alarcon et al., 2009; Kotov et al., 2010; Le Vigouroux et al., 2017; Mikolajczak et al., 2017). Second, co-parenting disagreement affected every burnout facet. Not feeling oneself to be on the same page as one’s spouse represented a risk factor in every parenting setting. This echoes previous research highlighting that conflicts in the caregiving family system put people at risk of burnout (Almberg et al., 2000). This is especially important when providing care as a parent, where the partner’s support is essential. These results also complete previous research highlighting the deleterious impact of poor quality couple relationships on burnout in a caregiving context (Lindström et al., 2011; Riva et al., 2014). Some could also argue a reverse relationship, in other words, that an increase of burnout symptoms has a deleterious impact on the relationship with the spouse, with direct consequences on co-parenting (dis)agreement. This hypothesis must be assessed in future research but it is more than likely that feedback loops occur between poor quality couple relationships and burnout.

Regarding descriptive variables, the most surprising result lies in the unexpected risk factor of being a father with a CSN. Both for EE and LPA – with a tendency for ED – being a father appeared to increase burnout. This adds confusion to an already disputed literature about the impact of gender on PCg burnout. Where some studies showed increases of burnout symptoms among mothers (Jaramillo et al., 2016), others did not consistently find such a gender difference (e.g., Lindahl Norberg, 2007). Yet, there was no evidence of males experiencing more burnout symptoms than women in a caregiving context. In the present study, those associations did not appear with Pearson correlations but did manifest when computed with other predictors. This might suggest an interaction between the gender and several variables, as previously shown when comparing fathers and mothers (Lindahl Norberg et al., 2014). Further research should investigate why fathers differ from mothers when being PCg.

With regard to other descriptive variables, such as education or age, these were rather small and inconsistent predictors of burnout. Unexpectedly, a positive correlation appeared between education level and ED for parents with no CSN. To our knowledge, previous burnout literature did not report such relationship in the past. Such effect – if appearing in other studies – should further be investigated.

Including consequences specific to the parent-caregiving context allowed us to better understand the EE of PCg. Indeed, three statements were related to more EE: (1) perceiving that the issue of the child has an impact on the family organization, (2) having preoccupations about the issue of the child, and (3) feeling that the issue resulted in giving up important things. Perceiving that the issue of the child has an impact on the family organization represents a risk factor only for EE. This may reflect both the absence of family organizational need – meaning a low disruption of the family – and the perfect integration of the special needs into the family – meaning that they do not represent an additional stress. In both cases, the organization of the family remains stable and demonstrates an absence of problems. If everything is going well in the family and for the parent, EE should diminish. Having constant preoccupations regarding the child’s issue also plays a role by the constant tension it puts on the parent. This constant tension represents a chronic stressor and thus facilitates EE. Preoccupation has already been shown as a risk factor among caregivers of psychiatric patients, increasing worry about the care recipient being related to higher burnout (Cuijpers and Stam, 2000). This also calls for further research about the importance of ruminative processes – as it has been studied in professional burnout (Brackett et al., 2010). Having preoccupations also impacts ED. More preoccupations lead to more ED with one’s children. The variable of constant care/attention – though significantly correlated with burnout – does not appear in the regression models. This is probably due to a widely shared variance and conceptual relatedness with the present variable of constant preoccupation. Perceiving having given up life yielded low response rates (8%), probably due to the extreme content. On the contrary, the variable assessing giving up important things appeared more common among PCg (30.9%). It could also be a key element in understanding PCg burnout because it emphasizes the aspect of self-sacrifice possibly involved in being a PCg. As for co-parenting disagreement, it could be both an antecedent and a consequence of burnout. As an antecedent, co-parenting disagreement could be a constant cognitive stressor. Parents would always feel they had abandoned important things in their lives only for the well-being of the child. Paired with ruminations, this might be a risk factor of EE. The feeling that the issue of the child has taken important things away from the parent may cause mixed feelings, including resentment against the child, which could then lead to ED with the child. However, such cognitions and feelings could also be seen as a biased observation of one’s life, everything looking darker when experiencing burnout. Altogether, when comparing comorbidity and multiple CSN groups with the no-CSN group, burnout was found to be significantly less important among the latter. Nevertheless, when considered in a regression with subjective variables such as the perceived consequences of being a PCg, these subjective consequences seemed to be more related to burnout than the presence of comorbidity or multiple CSN. The effect seems to move from objective indicators to subjective indicators. This might suggest that the subjective perception of one’s situation is a central element in understanding the consequences of caregiving. This is consistent with the literature on informal caregivers’ burnout: objective factors (e.g., number of hours per week, presence of comorbidity) play a role in the understanding of burnout (Yan, 2014), but perception remains one of the strongest indicators (Truzzi et al., 2008). Thus, it might not be the objective demands but rather the parent caregiver’s perception of his or her well-being that matters. For example, where some people may find it normal - and not very tedious - to carry out certain tasks, others will have a great deal of distress with consequences for their well-being. On the contrary, parents who are more resilient because they find more meaning in their parent-caregiving action, may be able to reduce this subjective hardship and its subsequent impacts. Previous studies have highlighted the importance of subjectivity and idiosyncratic perception in the caregiving context. This subjectivity has widely been studied in the informal caregiving literature in the form of *subjective burden*, the subjective weight of being a caregiver (Chiao et al., 2015). Studies have consistently shown a positive relationship between subjective burden and burnout among informal caregivers (e.g., Truzzi et al., 2008; Cill Akinci and Pinar, 2014). In the professional literature, similar findings exist with related variables: the accumulation of subjective stressors is a better predictor of professional burnout than the quantity or type of objective stressors themselves. Thus, rather than the objective stressors, it is the perception of such stressors that is consistently found to have the greatest impact on burnout reactions. The present study makes no exception because it is through the lenses of the person’s frame of reference that stressors may (or not) generate the idiosyncratic experience of feeling burned out.

### Limitations

The present study also has some limitations. One of the main limitations is its cross-sectional design. Though the sample is relatively large, the studied relationships are all correlational and so no clear conclusions of causality can be drawn. Conceptually, this design does not allow to test the bi-directionality of variables that could be conceived as both antecedents and consequences of burnout. Further qualitative studies should investigate the chronological progression of several of the studied variables, for example, to clarify if the feeling of having to give up important things due to the special issue of the child causes burnout or results from it. A second limitation lies in the broad categories of the issues encountered by the parents and their child(ren). Indeed, one does not really know the specific mental or physical problems that the children were going through. Future studies should better assess the specific issues encountered since these may represent very different realities for children and parents. In a similar vein, the third limitation of this study deals with the self-reported nature of the questionnaire. Because there is no control of the diagnostics of the children, there is a possibility of false reporting. Respondents could have reported having a child with a behavioral or emotional disorder just because they have the feeling of this without any reliable or clinical assessment of it. Although further research should control this, it should not undermine the reachability of the purposively large spectrum of PCg interrogated. More globally, it is not excluded that the recruitment methods used may have affected the sample representativeness. The use of platforms such as Prolific for research purposes in psychology is growing but might lead to biases associated with the remuneration participants perceive. Conversely, the common social media and snowball recruitment methods used could also lead to biases. Though it does not guarantee a bias-free study, the combination of these recruitment procedures aimed at counterweighting each method’s downsides and reducing overall biases in the sample. Finally, a fourth limitation is the relatively small PCg sample size. Although up to 19% of the total sample was categorized as PCg, it represented 175 people. Even if this sample size is sufficient for some analyses, it could also have led to possible false negative results. Indeed, the absence of difference between parents having one CSN and those with no-CSN regarding burnout measures could be due to a lack of statistical power. This difference – although not significant – appeared small for EE (Cohen’s *d* = 0.24). A G^∗^Power computation (Faul et al., 2007) indicated that such difference would have needed 280 PCg and 1168 parents with no-CSN to reveal a statistically significant difference – nearly 40% more participants.

### Generalizability and Implications

The present results on PCgs may give insights to both parental and informal caregiving burnout literature. First, the subjective impact of having a CSN could also be investigated among people becoming parents or parents having an additional child. In the present study, the impact of having a CSN was the main focus of attention but this effect could be investigated for every parenting setting. For example, feelings of having to give up important things due to having a child could further be studied among parents of typical children to check whether this might also affect parental burnout. Even if it is more acceptable to disclose that one has to give up things because one has a child with special needs, this feeling could also be present among parents of typical children. The present results are also consistent with previous studies regarding the importance of co-parenting (dis)agreement and neuroticism in the occurrence of parental burnout. Second, the present study gives insights for the wider informal caregiving literature on burnout. Our results bear similarities with research on the subjective burden experienced by caregivers, showing that this burden is a major predictor of EE. It is also the first study to highlight a relationship between neuroticism and burnout measures among informal caregivers. Finally, the results about the similar impact of variables on both parents with and without CSN also suggest that PCg are still parents, so each of these fields of research could learn from the other.

## Author Contributions

PG and EZ designed the hypotheses and data analyses and critically discussed the results and wrote the original paper. PG managed the data and ran the statistical analyses.

## Conflict of Interest Statement

The authors declare that the research was conducted in the absence of any commercial or financial relationships that could be construed as a potential conflict of interest.

## Footnotes

1 1 Items EE1 to EE8 and PA1 to PA6 Copyright 1981 Christina Maslach and Susan E. Jackson. All rights reserved in all media. Published by Mind Garden, Inc., www.mindgarden.com Altered with permission of the publisher.

## Abbreviations

CSN: child(ren) with special needs
PCg: parent-caregiver
